# Forging the Future: B Cell Activating Factor’s Impact on Nephrotic Syndrome

**DOI:** 10.21315/mjms2024.31.6.5

**Published:** 2024-12-31

**Authors:** Astrid Kristina Kardani, Loeki Enggar Fitri, Nur Samsu, Krisni Subandiyah

**Affiliations:** 1 Doctoral Program in Medical Sciences, Faculty of Medicine, Universitas Brawijaya, Malang, Indonesia; 2 Nephrology Division, Department of Paediatric, Faculty of Medicine, Universitas Brawijaya, Dr. Saiful Anwar General Hospital, Malang, Indonesia; 3 Department of Clinical Parasitology, Faculty of Medicine, Universitas Brawijaya, Dr. Saiful Anwar General Hospital, Malang, Indonesia; 4 Nephrology Division, Department of Internal Medicine, Faculty of Medicine, Universitas Brawijaya, Dr. Saiful Anwar General Hospital, Malang, Indonesia

**Keywords:** nephrotic syndrome, BAFF, TLR, B cell

## Abstract

Nephrotic syndrome is the most common glomerular disease in children. While the exact pathogenesis of nephrotic syndrome is not fully understood, recent research has shed light on some of the underlying mechanisms involved in it. Improvement by B cell depletion therapy using antiCD20 in nephrotic syndrome has led to a paradigm shift from immunoinflammatory disease influenced by T cell dysregulation to B cell involvement in the pathogenesis of nephrotic syndrome. The expression of the B cell activating factor (BAFF), an essential cytokine for the maturation and differentiation of B lymphocytes, in the podocytes of paediatric patients with nephrotic syndrome is known to be associated with worse renal outcomes. The pro-inflammatory cytokines and pathogenic antibodies produced by B cells allegedly cause podocyte injury leading to proteinuria due to effacement of foot processes. Considering the role of the BAFF in B cell proliferation and antibody production, BAFF signalling is a potential target for development as targeted therapy in nephrotic syndrome. Nevertheless, there is limited research regarding the role of BAFF in nephrotic syndrome, and the exact mechanism of BAFF involvement in the pathogenesis of nephrotic syndrome is still unknown. This review discusses the role of the BAFF in the pathogenesis of nephrotic syndrome and highlights the gap of knowledge for future research.

## Introduction

Nephrotic syndrome has become the most common glomerular disease in children, yielding an incidence of one to three cases per 100,000 children (1). Nephrotic syndrome, which is characterised by massive proteinuria that causes hypoalbuminemia, oedema, and hyperlipidaemia, is believed to be an immunoinflammatory disease influenced by T cell dysregulation (2). According to its definition, nephrotic syndrome is a condition that is not caused by autoantibodies or immune complexes; thus, in many cases of focal segmental glomerulosclerosis (FSGS) and minimal change disease (MCD), most immune complexes or cellular inflammations are not found, and the involvement of the immune system in nephrotic syndrome is likely through factors secreted by immune cells. B cell depletion therapy by antiCD20 treatment in nephrotic syndrome patients recently showed good results, leading to a paradigm shift with regard to the involvement of B cells in the pathogenesis of nephrotic syndrome (3). Subsequently, many case reports and clinical trials emerged in relation to B cell depletion therapy maintaining long-term remission in both children and adult nephrotic syndrome patients (4, 5).

The expression of the B cell activating factor (BAFF), which is an essential cytokine for the maturation and differentiation of B lymphocytes, in the podocytes of paediatric patients with nephrotic syndrome is known to be associated with worse renal outcomes. The interaction between the Toll-like receptor (TLR), Type 1 interferon (IFN), and BAFF forms an amplification effect that triggers antibody production and promotes dysregulation of B cell activation, which then produces immune complexes and cytokines that trigger kidney tissue injury, especially podocytes (6–11).

Although the role of B cells is well known in other autoimmune diseases, studies on their involvement in nephrotic syndrome are limited. With the high recurrence rate of corticosteroid therapy, the discovery of the BAFF’s role in nephrotic syndrome will disclose new therapeutic opportunities that are effective in reducing the recurrence rate in nephrotic syndrome patients. In this review, we discuss the BAFF’s involvement in the pathogenesis and treatment of paediatric nephrotic syndrome based on existing literature.

## TLR Involvement in the Pathogenesis of Nephrotic Syndrome

**Figure 1:**
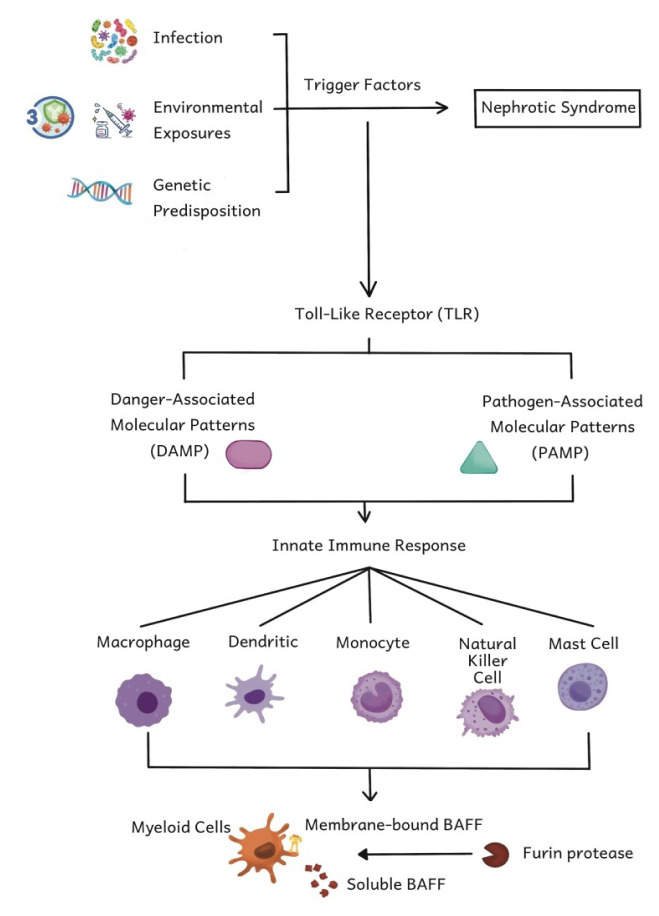
TLR and BAFF in the pathogenesis of nephrotic syndrome

TLR are innate immune receptors recognising the structural components of microorganisms by detecting danger-associated molecular patterns (DAMP) and pathogen-associated molecular patterns (PAMP) (6) as shown in Figure 1. In many autoimmune diseases, TLRs serve as innate immune receptors identifying structural components or microorganisms and triggering a direct pro-inflammatory response. Infection was once thought to contribute to the onset of idiopathic nephrotic syndrome. A study found that 70% of relapse cases are preceded by an upper respiratory tract infection. Respiratory syncytial virus, varicella zoster virus, influenza virus, parainfluenza virus, and adenovirus were identified as possible causes through viral cultures. Upper respiratory tract infections have a strong correlation with proteinuria in patients with relapsing nephrotic syndrome. The recognition of antigen epitopes by PAMPs triggers TLR activation, which then induces B cell differentiation and immunoglobulin production via the NF-κB pathway (6, 7).

TLR via PAMPS and DAMP will initiate native and adaptive immune responses through complement and inflammatory cells, such as macrophages. TLR activation triggers the release of the NF-κB transcription factor, which will lead to the production of inflammatory mediators, such as Il-1, IL-1, IL-6, IL-12, and TNF-α, and cause damage to the glomerulus. Native immune cells then transform into a cycle of antigenspecific reactions. This transformation involves several possible mechanisms, such as the regulation of natural autoimmunity, and epitope changes, molecular mimicry, or the phenomenon of autoantigen complementarity. TLRs also need to activate adaptive immune cells through APCs, which trigger B cell activation, CD4 helper differentiation, and antibody production. Antibodies cause the trapping or formation of immune complexes locally, which will activate TLRs and complement the components of the native immune system (6, 7).

## Current Immunopathogenesis of Nephrotic Syndrome

Idiopathic nephrotic syndrome is mainly caused by intrinsic kidney diseases like MCD and FSGS. Factors, such as infection, environmental exposure, and genetic predisposition, can trigger the emergence of nephrotic syndrome (as shown in Figure 1). Almost 50% of cases are triggered by infections, particularly in the upper respiratory tract (12). The pathogenesis of idiopathic nephrotic syndrome is still not clearly understood, but some pieces of evidence indicate a possible involvement of the immune system in the pathogenesis of nongenetic idiopathic nephrotic syndrome. The involvement of the immune system in the pathogenesis of nongenetic idiopathic nephrotic syndrome is suggested by the efficacy of various immunosuppressant approaches and the important role of prednisone in its treatment (3, 4). However, the mechanism of immune system dysregulation that disrupts the glomerular filtration barrier and causes proteinuria is still uncertain. The main diagnostic feature of nephrotic syndrome is foot process effacement. Shalhoub (13) hypothesised that idiopathic nephrotic disease is characterised by the dysregulation of systemic T cells, which results in the production of circulating mediators that can modify the podocyte structure and trigger foot process fusion. However, in 2006, Pescovitz et al. (3) reported a case of a posttransplant FSGS recurrence that went into remission after receiving B cell depletion therapy in the form of rituximab, which was originally meant to treat posttransplant lymphoproliferative disease in a seven-year-old kid. Following that, the involvement of B cells in the development of nephrotic syndrome became increasingly clear. Numerous clinical studies have demonstrated favourable clinical results with B cell depletion therapy for the management of long-term remission in patients with nephrotic syndrome, including both children and adults. Studies showed the therapeutic efficacy of anti-CD20 antibodies in maintaining or inducing the remission of nephrotic syndrome, even with the discontinuation of immunosuppressant therapy (4, 5, 14).

Various mechanisms are generally believed to produce circulating factors that can affect podocytes and disturb the glomerular filtration barrier. In contrast to other glomerular diseases, INS is not usually considered an immune-mediated disease due to a lack of evidence. However, in 1998, Dantal et al. (15) discovered permeability factors that can bind with immunoglobulins and cause albuminuria. This antibody is estimated to be found in 29% of both adult and children nephrotic syndrome patients. This finding was later supported by Harinouchi et al. (16), who found antinephrin IgG at the initial esentation in 50% of paediatric nephrotic syndrome patients in Japan. The role of B cells independent of antibodies recently emerged in various disease models B cells are known to produce cytokines that can inhibit or trigger inflammatory responses. Cytokines secreted by B cells are essential for the formation of ectopic lymphoid follicles and lymph nodes and can enhance T cell responses (9). In their study, Kim et al. showed that the local activation of B cells in the glomerulus can produce sufficient IL-4 to trigger proteinuria due to foot process effacement (17). The exact role of IL-4 in causing damage to the glomeruli is still a matter of debate, but it is known to play a role in several cases of systemic lupus erythematosus (SLE) and immunoglobulin A (IgA) nephropathy. Th2 cells can be released by interleukin-4, which is associated with MCD, a condition strongly linked to atopy (18).

## BAFF as a Regulator of B cell Activation

**Figure 2:**
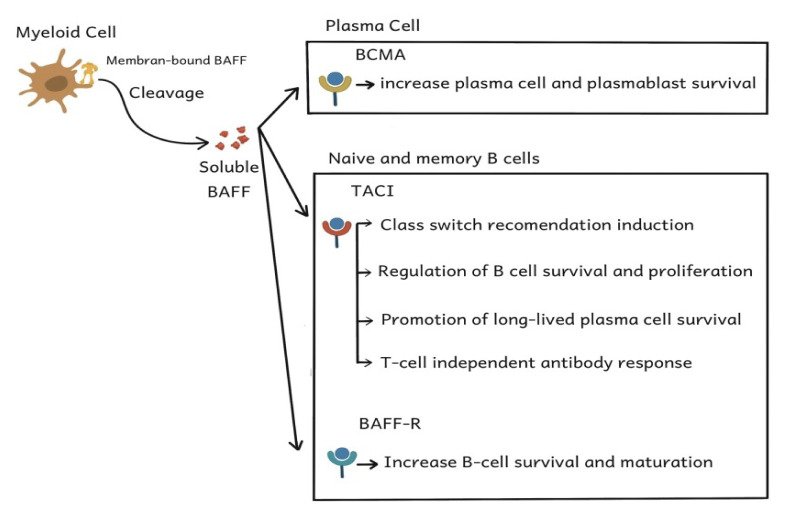
Myeloid cells as the main producers of BAFF

The B cell ontogenecity is regulated via the BAFF system comprising BAFF, a proliferation-inducing ligand (APRIL). Its receptors are the B cell maturation antigen (BCMA), BAFF-Receptor (BR3/BAFF-R), and transmembrane activator and CALM interactor (TACI) (as shown in Figure 2). The BAFF and APRIL, which are members of the TNF family, are essential cytokines that trigger the B cell proliferation, survival, and maturation via the BAFF-R, TACI, and BCMA receptors. The human BAFF gene has multiple exons. Exon 1 codes for the transmembrane domain and the surrounding region, while exon 2 codes for the processing part. Exons 3–6 are responsible for the TNF homologue domain that binds to the receptor (19, 20). As shown in Figure 2, the BAFF cytokines are mainly produced from myeloid cells, which include several types of immune cells, such as dendritic cells, neutrophils, monocytes, and macrophages. This happens in response to the expression of TLR, types I and II interferon, IL-10, and granulocyte colony-stimulating factor (GSCF). The BAFF is a type II membrane-bound protein that can secrete trimeric ligands. Following cleavage by furin protease, the BAFF is released into the circulation as a soluble, biologically active 17 kDa protein. The BAFF will then bind to the receptors, each of which has a different expression pattern depending on the level of B cell development and is associated with different functions (20–22).

At the transitional B cell level, BAFF-R is the primary receptor found on both naive and memory B cells; BCMA is the main receptor found on long-term plasma cells; and TACI is the primary receptor found on the B cells in the marginal zone of short-term plasma cells. Myeloid cells, such as mast cells, macrophages, dendritic cells, monocytes, and neutrophils, are the primary BAFF producers under physiological conditions. However, the BAFF expression can increase due to stimulation from TLR activation, pro-inflammatory cytokines, and lipopolysaccharides from bacteria (20–22).

## BAFF in Pathogenesis of Nephrotic Syndrome

The most important indication of the pathogenic role of B cells in nephrotic syndrome is explained by the emerging therapeutic response to therapies that are directly or indirectly affecting the B cell function. High doses of prednisolone interfere with B cell proliferation and differentiation and IgG production. Calcineurin inhibitors, such as cyclosporine and tacrolimus, are commonly used to prevent steroid toxicity and modify the balance of differentiation, apoptosis, and proliferation of B cells in idiopathic nephrotic syndrome. Mycophenolate, which is a steroid sparing agent in nephrotic syndrome, also inhibits the proliferation of B or T lymphocytes and interferes with antibody production. This suggests that appearing effects are related to the duration of the B cell depletion that occurs, explaining why remissions can be maintained in nephrotic syndrome patients who receive rituximab therapy (3, 23–26). In autoimmune diseases, TLR activation can induce type I IFN production, increase the BAFF breakdown from myeloid cells to soluble BAFF, and increase B cell receptor signalling and antibody production. The interaction between TLR, type 1 IFN, and BAFF forms an amplification effect that triggers antibody production and encourages the dysregulation of B cell activation, which then produces immune complexes and cytokines that trigger kidney tissue injury, especially podocytes (6, 7).

Disturbances in the balance of BAFF or APRIL or their receptors are related to autoimmune diseases in human and mouse models. Elevated BAFF levels are known to be associated with several autoimmune diseases, including multiple myeloma, Sjogren’s syndrome, SLE, rheumatoid arthritis, and Hodgkin’s and nonHodgkin’s lymphoma (10, 20). In their study, Kamhieh-Milz et al. (27) showed that the BAFF levels in the serum of patients with active immune thrombocytopenic purpura significantly increase. In this patient, therapy using glucocorticoids is known to reduce the BAFF levels but does not reduce the APRIL levels. Based on the performed in silico studies, five transcription factors for the glucocorticoid receptor were found to be located in the BAFF promoter region, but not in APRIL (27).

The expression of BAFF and its receptors is related to severe lupus nephritis and thought to be a crucial factor in renal involvement in SLE patients. In their work, Cao et al. (23) found that BAFF plays a role in the pathogenesis of IgA nephropathy by increasing the activation of the NF-κB pathway. The BAFF in CKD patients with COVID-19 is also known as an independent risk factor for mortality in the hospital and within 30 days. The role of BAFF in nephrotic syndrome is still unknown, but recent studies showed that increased BAFF in patients with nephrotic syndrome is associated with worse clinical outcomes. In their study, Han et al. (28) showed an increase in the BAFF expression in patients with membranous nephropathy. High BAFF levels are also presented to be a hallmark of minimal change nephrotic syndrome in adults (28). The BAFF expression profile in this study appears similar to that of other autoimmune diseases. This study further strengthens the role of B cells in the pathogenesis of nephrotic syndrome and opens opportunities for the role of BAFF as a therapeutic target for nephrotic syndrome (29).

## BAFF as a New Therapeutic Target in Nephrotic Syndrome

Targeted therapy is a treatment approach commonly used to manage glomerular diseases. This therapy targets specific cellular or molecular processes involved in disease development or progression. The goal of this therapy is to reduce the adverse effects of traditional immunosuppressive therapy, which is currently the first-line therapy for nephrotic syndrome, despite having a high recurrence rate (30). Numerous studies have discovered that therapy with antiCD20 monoclonal antibodies can induce and uphold remission, making B cells a new therapeutic target for nephrotic syndrome. A therapy that inhibits the B cell activity by causing constant B cell depletion has been developed as a treatment option for challenging cases of nephrotic syndrome (31). Several therapies are currently being developed to target molecules that are participating in B cell homeostasis. Considering the role of BAFF in B cell proliferation and antibody production, BAFF signalling is a potential target for development as targeted therapy in nephrotic syndrome (30, 32).

## Challenge for Future Research

**Figure 3:**
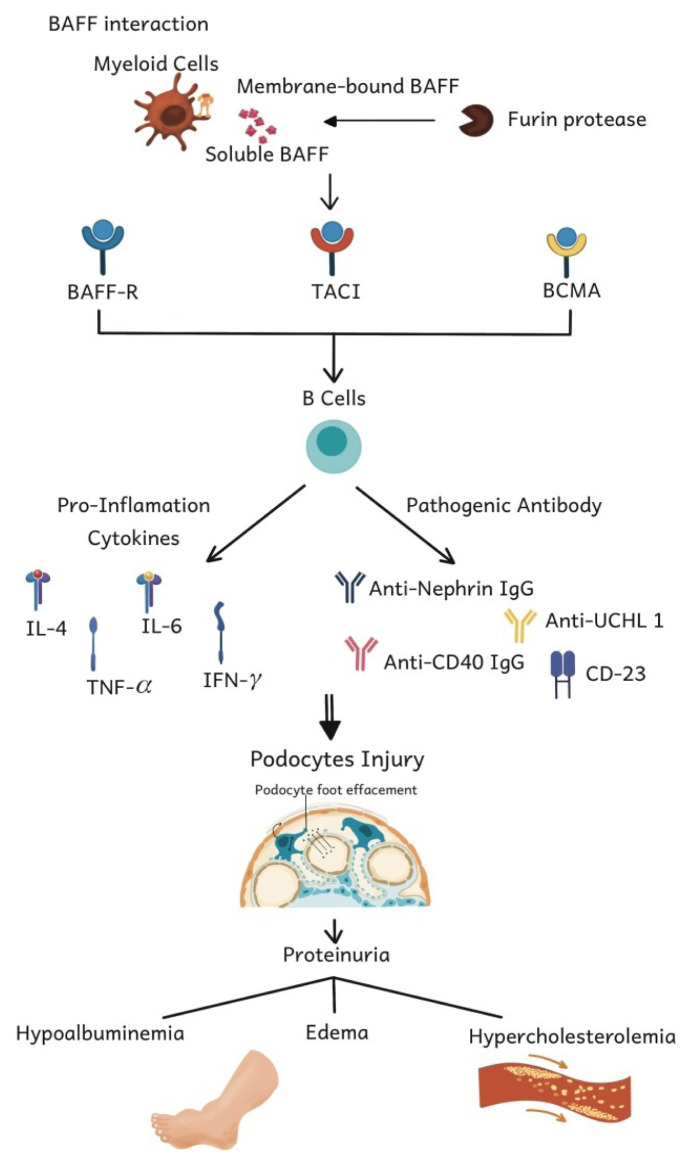
BAFF involvement in the immunopathogenesis of nephrotic syndrome

There is limited research regarding the role of BAFF in nephrotic syndrome. Although there is evidence that BAFF contributes to the pathogenesis of nephrotic syndrome, the exact mechanism remains unknown. Due to the complex immune responses involved, it is often difficult to distinguish between the roles of T and B cells in nephrotic syndrome because they influence each other (9). As various studies have attempted to determine the exact mechanism of BAFF involvement in nephrotic syndrome in both human and animal models, this is a vast research field that requires further exploration (as shown in Figure 3).

In SLE patients, the BAFF levels are known to increase, resulting in the development of a recombinant antibody therapy that targets soluble BAFF. This therapy has been proven to provide significant benefits in SLE and lupus nephritis (LN) patients (33). Belimumab, a monoclonal antibody that binds to BAFF, is the first biological medicine approved by the Food and Drug Administration as a therapy for SLE and LN. Belimumab intrudes with B cell survival and causes its depletion (10, 31, 32). In primary glomerular disease, the use of a BAFF antagonist in the form of belimumab reduces proteinuria in primary membranous nephropathy (34). The plasma levels of BAFF correlate with the severity of histological damage in IgAN (23). Although there have been many studies on antiBAFF in autoimmune or glomerular disease, there is only one clinical study of antiBAFF in nephrotic syndrome. This was conducted on five children with frequent relapse nephrotic syndrome (FRNS) by administering belimumab injections for 12 months, along with the tapering or discontinuation of prednisone. The study revealed that belimumab therapy is well tolerated in paediatric FRNS. However, the number of subjects was too small to draw any significant conclusions. Moreover, the high therapy costs outweighed the benefits that were not yet clearly visible, leading to the discontinuation of the study (35).

The targeted therapy approach using BAFF in nephrotic syndrome also needs further research because the current study failed to give any conclusion due to limited subjects. Investigating which patient group would benefit from the inhibition of BAFF and APRIL could facilitate precision medicine for patients.

## Conclusion

Several studies suggested that BAFF may have a significant role in the pathogenesis of nephrotic syndrome. These studies prompted further research into the role of B cells in this condition. Despite numerous studies, the mechanism by which BAFF is involved in nephrotic syndrome remains unclear. Consequently, B cell activation by BAFF and the pathways involved in this process are being extensively studied as potential therapeutic targets for patients with nephrotic syndrome.
